# Tumor-Associated Macrophages as Target for Antitumor Therapy

**DOI:** 10.1007/s00005-017-0480-8

**Published:** 2017-06-28

**Authors:** Katarzyna Sawa-Wejksza, Martyna Kandefer-Szerszeń

**Affiliations:** Department of Virology and Immunology, Institute of Microbiology and Biotechnology, Maria Curie-Skłodowska University, Akademicka 19, 20-033 Lublin, Poland

**Keywords:** Tumor-associated macrophages, TAMs, Cancer therapies, Immunotherapy, Tumor

## Abstract

It is well known that the microenvironment of solid tumors is rich in inflammatory cells that influence tumor growth and development. Macrophages, called tumor-associated macrophages (TAMs), are the most abundant immune cell population present in tumor tissue. Several studies have demonstrated that the density of TAMs is associated with a poor prognosis and positively correlates with tumor growth. Several studies have proved that TAMs may activate and protect tumor stem cells, stimulate their proliferation as well as promote angiogenesis and metastasis. Furthermore, TAMs-derived cytokines and other proteins, such as CCL-17, CCL-22, TGF-β, IL-10, arginase 1, and galectin-3, make a significant contribution to immunosuppression. Since TAMs influence various aspects of cancer progression, there are many attempts to use them as a target for immunotherapy. The numerous studies have shown that the primary tumor growth and the number of metastatic sites can be significantly decreased by decreasing the population of macrophages in tumor tissue, for example, by blocking recruitment of monocytes or eliminating TAMs already present in the tumor tissue. Moreover, there are attempts at reprogramming TAMs into proinflammatory M1 macrophages or neutralizing the protumoral products of TAMs. Another approach uses TAMs for anticancer drug delivery into the tumor environment. In this review, we would like to summarize the clinical and preclinical trials that were focused on macrophages as a target for anticancer therapies.

## Characteristic of Tumor-Associated Macrophages

The importance of the tumor microenvironment in the initiation and promotion of cancer has been increasingly recognized nowadays (Bissell and Hines 2011). It is well known that the microenvironment of solid tumors is rich in inflammatory cells and macrophages, called tumor-associated macrophages (TAMs), are the most abundant immune cell population in tumor tissue (Noy and Pollard 2014). Moreover, the tumor-associated immune cells very often differentiate into suppressive cells, which support tumor growth and development (Cabarcas et al. 2011) and lose the ability to eliminate cancer cells.

Various mouse and human studies have shown that monocytes are recruited into tumors by chemokines secreted by malignant and stromal cells. Tumor-derived chemokine CCL-2 is a monocyte-chemotactic protein and its high level correlates with increased numbers of TAMs in tumor tissue and a poor prognosis. Other chemokines, such as CCL-3, CCL-4, CCL-5, CCL-7, CCL-8, CXCL-12, and cytokines, including vascular endothelial growth factor (VEGF), platelet-derived growth factor (PDGF), macrophage colony stimulating factor (M-CSF), and interleukin (IL)-10, are also reported to promote macrophage recruitment (Allavena et al. 2008; Murdoch et al. 2008). In the tumor site, monocytes differentiate into TAMs depending on the local microenvironments.

Macrophages are extremely plastic cells. In mature adults, they may differentiate into proinflammatory classical (M1) or suppressive alternatively activated (M2) macrophages depending on the local microenvironment. The M1 macrophages differentiate in the presence of helper Th1 cytokines or upon activation of Toll-like receptors (TLRs) and they are involved in Th1 responses to pathogens. M1 macrophages can be characterized as cells that produce cytokines such as IL-12, IL-1, IL-6, tumor necrosis factor (TNF)-α, reactive oxygen species (ROS), and nitric oxide (NO) and exhibit increased expression of the MHC II class (Sica and Mantovani 2012). On the other hand, Th2 cytokines such as IL-4, IL-10, and IL-13 induce polarization into the M2 macrophages participating in Th2 immune response such as humoral immunity, wound healing, and tissue remodeling (Gordon and Martinez 2010).

In tumor tissue, TAMs are composed of several distinct populations that share features of both M1 and M2 macrophages; however, most studies have shown that TAMs are anti-inflammatory and correlate with a poor prognosis (Qian and Pollard 2010). It seems likely that classification of TAMs should be related mainly to their function such as metastasis-promoting, angiogenic, and immunosuppressive macrophages. Antibodies to the glycoprotein CD68, CD14, CD312, CD115, HLA-DR, and CD16 have been used for macrophage classification. Other proteins such as CD163 (hemoglobin-scavenger receptor), MRC1, CD206 (mannose receptor), and TIM-3 (T-cell immunoglobulin and mucin domain containing protein-3) have been used for classification of TAMs (Heusinkveld and van der Burg 2011). It has been proved that TAMs support growth and directly participate in various aspects of tumor development. Moreover, TAMs stimulate cancer cell proliferation, activate tumor stem cells, and promote metastasis (Mantovani and Sica 2010). TAMs influence the tumor microenvironment by secreting matrix metalloproteinases (MMPs) or IL-1β and induce vascularization of tumor tissue by producing VEGF, PDGF, and transforming growth factor (TGF)-β (Dirkx et al. 2006). They also release chemoprotective factors, for example, cathepsin b and milk-fat globule EGF-VIII, which promote tumorigenicity of cancer stem cells and induce anticancer drug resistance (Bruchard et al. 2013; De Palma and Lewis 2013; Jinushi et al. 2011). Furthermore, several studies have reported that TAM-derived cytokines and other proteins, such as CCL-17, CCL-22, TGF-β, IL-10, arginase 1, and galectin-3, make a significant contribution to immunosuppression (Qian and Pollard 2010; Biragyn and Longo 2012).

Since TAMs influence various aspects of cancer progression, they can serve as a target for clinical therapy. Moreover, accumulating studies have demonstrated that the density of TAMs is associated with a poor prognosis and positively correlates with the proliferation of cancer cells in several tumors, such as breast, endometrial, and renal cancer (Heusinkveld and van der Burg 2011). There are numerous studies reporting attempts at blocking infiltration of macrophages into tumor tissue or reducing the population of macrophages, which significantly decreased primary tumor growth and the number of metastatic sites. In this review, we would like to summarize the clinical and preclinical trials that were focused on macrophages as a target for anticancer therapy.

## Macrophage-Associated Therapies

The regular treatment for cancer patients is aggressive surgery of tumor tissue supported with radiochemical or hormonal therapy. However, none of these treatments is tumor specific and all of them have strong side effects. Therefore, increasing numbers of therapies focused on mobilization and strengthening the immune system to recognize and eliminate cancer cells is currently being tested in preclinical and clinical trials. One of these approaches is to use monocytes and macrophages as a target for immunotherapy.

There are many potential antitumor therapies involving TAMs (Tang et al. 2013). Attempts have been undertaken to decrease the population of protumoral macrophages in tumor tissue, for example, by blocking monocyte recruitment or eliminating TAMs already present in the tumor tissue. Furthermore, there are attempts at reprogramming TAMs into proinflammatory M1 macrophages or neutralizing the protumoral products of TAMs. Another approach uses TAMs for anticancer drug delivery into the tumor environment. Antitumor strategies that target TAMs are summarized in Table 1.

**Table 1:** Anti-tumor strategies that target tumor-associated macrophages (TAMs)

Treatment	Type of study	References
Blocking the monocytes recruitment into tumor tissue		
siRNA silencing the CCL2 expression	In vivo; mice; breast cancer	Fang et al. (2016)
Anti-CCL-2 antibody	In vivo; mice; glioma, ovarian, prostate, colon cancer	Loberg et al. (2007), Moisan et al. (2014), Popivanova et al. (2009), Zhu et al. (2011)
	Clinical study; phase 1, 2	Brana et al. (2015), Pienta et al. (2013), Sandhu et al. (2013)
CCR2 inhibitor	Clinical study; phase 1b	Nywening et al. (2016)
Anti-IL-6 antibody	Clinical study; phase 1, 2	Angevin et al. (2014), Coward et al. (2011), Karkera et al. (2011), Rossi et al. (2010)
Anti-CSF-1 antibody	In vivo; mice; breast cancer	Hollmén et al. (2015), Paulus et al. (2006), Ries et al. (2014)
CSF-1R inhibitors	In vivo; mice; breast, pancreas, ovarian cancer	Hollmén et al. (2015), Kubota et al. (2009), Mitchem et al. 2013, Moughon et al. (2015)
FLT3 inhibitors	In vivo; mice; glioblastoma, lung, melanoma, cervical, breast cancer	Kim et al. (2014), Manthey et al. (2009), Ngiow et al. (2015), Pyonteck et al. (2013), Sluijter et al. (2014), Stafford et al. (2016), Strachan et al. (2013)
	Clinical study; phase 1, 2	Butowski et al. (2016)
CXCR4 inhibitors	In vitro study	Beider et al. (2014)
	In vivo; mice; melanoma, breast cancer	Boimel et al. (2012), Hughes et al. (2015), Mota et al. (2016)
Decreasing the population of TAMs		
Bisphosphonates	In vivo; mice; melanoma, hepatocellular carcinoma, testis, lung cancer,	Hiraoka et al. (2008), Piaggio et al. (2016), Priceman et al. (2010), Zeisberger et al. (2006), Zhang et al. (2010)
Trabectedin	In vivo; mice; liposarcoma, fibrosarcoma, ovarian cancer	Germano et al. (2010, 2013)
	Clinical study; phase 2	Allavena et al. (2005), Germano et al. (2013), Gordon et al. (2016)
Legumain	In vivo; mice; colon, breast cancer	Lewēn et al. (2008), Liu et al. (2003, 2013), Xiang et al. (2008)
Anti-CD52 antibody (alemtuzumab)	In vivo; mice; ovarian cancer	Pulaski et al. (2009)
Anti-sr-a immunotoxin	In vivo; mice; ovarian, pancreatic cancer.	Neyen et al. (2013)
Anti-FRβ immunotoxin	In vitro study	Kurahara et al. (2012), O’Shannessy et al. (2015), Puig-Kröger et al. (2009), Shen et al. (2015)
	In vivo; mice; glioma	Nagai et al. (2009)
Anti-CD11b antibody	In vivo; mice; lung, squamous, colon, ovarian cancer	Ahn et al. (2010), Khan et al. (2015), Zhang et al. (2015)
Target delivery to MMR (CD206)	In vivo; mice; sarcoma	Zhan et al. (2014)
M2 macrophage-targeting peptide (M2pep)	In vitro study	Ngambenjawong et al. (2016)
Transformation of TAM into M1 proinflammatory macrophages		
Anti-CD40 antibody (CP-870.893, ChiLob7/4, dacetuzumab)	In vivo; mice; melanoma, glioma, pancreatic cancer	Alderson et al. (2013), Beatty et al. (2011, 2013), Buhtoiarov et al. (2006), Chowdhury et al. (2014), Johnson et al. (2015), Kosaka et al. (2014), Shi et al. (2015), Vonderheide et al. (2007, 2013a, b), Vonderheide and Glennie (2013)
	Clinical study; phase 1, 1b	Beatty et al. (2011, 2013), Chowdhury et al. (2014), Johnson et al. (2015), Vonderheide et al. (2007)
Thymosin-α	In vitro, cell cultures	Garaci et al. (2012)
	In vivo; mice; breast, ovarian cancer	Shrivastava et al. (2004, 2005)
Attenuated bacteria	In vivo; mice; breast, ovarian cancer	Galmbacher et al. (2010), Le et al. (2012), Lizotte et al. (2014)
	Clinical study; phase 1, 2	Le et al. (2012)
β-Glucan	In vivo; mice; breast, ovarian cancer	Albeituni et al. (2016), Liu et al. (2015)
	Clinical study; phase 1, 2	Albeituni et al. (2016), Kushner et al. (2014), Segal et al. (2016)
Sorafenib	Clinical study; phase 1,3	Deng et al. (2016), Llovet et al. (2008)
Metformin	In vivo; mice; lung, breast, pancreatic cancer	Ding et al. (2015)
Embelin	In vivo; mice; colon, pancreatic cancer	Dai et al. (2009), Peng et al. (2014), Wu et al. (2016)
Anti-MARCO antibody	In vivo; mice; melanoma, breast cancer	Georgoudaki et al. (2016)
siRNA	In vitro study	Kono et al. (2014), Ortega et al. (2016)
	In vivo; mice; lung cancer	Conde et al. (2015), Ortega et al. (2015)
miRNA	In vitro study	Cai et al. (2012), Su et al. (2016)
	In vivo; mice; liver, lung cancer	Chai et al. 2015; Squadrito et al. 2012
CpG ODN	In vivo; mice; melanoma, neuroblastoma, colon, lung cancer	Buhtoiarov et al. (2006), Chang et al. (2014), Huang et al. (2012), Le Noci et al. (2015), Lin et al. (2013), Shi et al. (2015), Shirota et al. (2012), Shirota and Klinman (2012)
HRG	In vivo; mice; pancreatic cancer	Rolny et al. (2011)
Inhibition of NF-κB pathway	In vivo; mice; melanoma, breast cancer	Connelly et al. (2011), Yang et al. (2014)
	In vitro study	Ortega et al. (2016)
Inhibition of STAT3 pathway	Clinical study; phase 2	Abdelraouf et al. (2016), Armstrong et al. (2016), Remon et al. (2016)
	In vivo; mice; melanoma, breast cancer	Cheng et al. (2003), Fujiwara et al. (2014), Kumar et al. (2016), Xin et al. (2009)
	In vitro study	Edwards and Emens (2010)

## Blocking the Monocyte Recruitment into Tumor Tissue

The recruitment of TAMs can be inhibited by blocking the monocyte-chemotactic chemokines and cytokines or their receptors by means of monoclonal antibodies (mAbs) or chemical agents. These therapies are currently being tested in preclinical and clinical trials (Germano et al. 2013; Ries et al. 2014).

The CCL-2 (MCP-1) is a C–C motif chemokine overexpressed in many solid tumors. Several studies have shown that the CCL-2 is used to recruit monocytes into an immunosuppressive tumor microenvironment. It has been proved that blocking of the CCL-2/CCR-2 axis decreased macrophage infiltration and reduced tumor growth (Lim et al. 2016). The use of the anti-CCL-2 antibody (Carlumab; CNTO88) was proved to block the growth of glioma, colon, prostate, and melanoma cancers in animal models (Loberg et al. 2007; Moisan et al. 2014; Popivanova et al. 2009; Zhu et al. 2011).

Clinical trials have been performed with the use of the anti-CCL-2 antibody (clinical trials NCT00537368, NCT00992186, and NCT01204996). However, a phase 2 study of carlumab in metastatic castration-resistant prostate cancer (CRPC) patients showed that this antibody was tolerated well but neither blocked the CCL-2/CCR-2 axis nor showed antitumor activity as a single agent in metastatic CRPC (Pienta et al. 2013). Similar results were observed in the study of Brana et al. (2015). They showed that carlumab in combination with four chemotherapy regimens for the treatment of patients with solid tumors was tolerated well, although no long-term suppression of serum CCL-2 or significant tumor responses was observed (Brana et al. 2015). However, according to Sandhu et al. (2013), carlumab was well tolerated with evidence of transient CCL-2 suppression and preliminary antitumor activity. Therefore, other studies are needed to clarify whether the promising effects observed in animal models can be successfully applied in treatment of human patients.

The other approach to decrease the action of CCL-2 is to block CCR-2. Pharmacological inhibitors and a humanized antibody that recognizes CCR-2 were examined. A CCR-2 inhibitor, PF-136309, in combination with FOLFIRINOX chemotherapy (oxaliplatin, irinotecan plus leucovorin and fluorouracil) was used in a phase 1b trial. Such therapy was safe and tolerable with objective tumor response (Nywening et al. 2016). Moreover, the efficiency of the humanized antibody specific for CCR-2 (MLN1202) is under clinical investigation (clinical trial NCT01015560). Another approach to target the CCL-2/CCR-2 axis is to use siRNA, which targets CCL-2 expression in vivo by a complex of siRNAs with tumor penetrating peptides by non-covalent calcium cross-linking. This complex silenced tumor growth and metastasis associated with reduced recruitment of M2 macrophages in a mouse breast cancer model (Fang et al. 2016).

Furthermore, the use of the anti-IL-6 antibody had a strong anticancer effect also by decreasing CCL-2, VEGF, and CXCL-12 (Chen and Chen 2015). Siltuximab (CNTO 328) is a chimeric murine-human antibody that was confirmed to neutralize IL-6 and proved to have an effect on a number of human malignancies. The influence of siltuximab on several solid tumors like prostate, ovarian, lung, and renal cancer was observed in several clinical trials. A preliminary studies showed that siltuximab was safe and well tolerated, but did not show a strong clinical effect as a monotherapy in an advanced stage of colorectal, ovarian, and pancreatic tumors (Angevin et al. 2014). However, a phase 1 study showed that siltuximab downregulated genes implicated in tumorigenesis in prostate cancer patients (Karkera et al. 2011) and stabilized the disease in more than 50% patients in a phase 1/2 study in metastatic renal cancer (Rossi et al. 2010). Furthermore, Coward et al. (2011) showed a significant decline in CCL-2, CXCL-12, and VEGF as well as the number of TAMs in tumor tissue in patients treated with siltuximab for 6 months. Therefore, further studies, in which combination therapy could be used, are promising and justified.

It was proved that targeting not only CCL-2 but also CSF-1 (M-CSF) improves chemotherapeutic efficacy, inhibits metastasis, and increases antitumor T-cell responses (Mitchem et al. 2013). CSF-1 is a chemokine involved in a wide range of biological processes. It influences proliferation, differentiation, and survival of many cell types including monocytes and macrophages. CSF-1 also stimulates the chemotactic activity of monocytes and macrophages. It was shown that G-CSF regulates the macrophage phenotype and is associated with poor overall survival in human triple-negative breast cancer (Hollmén et al. 2015). The G-CSF blockade in the 4T1 mammary tumor model promoted maturation of blood monocytes and TAMs and significantly reduced lung metastasis (Hollmén et al. 2015). Similar results were observed in the case of mouse osteosarcoma. The inhibition of M-CSF by both an antibody or a chemical inhibitor (Ki20227) significantly suppressed tumor angiogenesis and lymphangiogenesis (Kubota et al. 2009). Moreover, macrophage blockade using a CSF-1R inhibitor (GW2580) resulted in reduced infiltration of protumorigenic (M2) macrophages. It also reversed the vascular leakage underlying malignant ascites in late-stage epithelial ovarian cancer (Moughon et al. 2015). Combination chemotherapy (cyclophosphamide, methotrexate, and 5-fluorouracil) in human breast cancer xenografts grown in immunodeficient mice was more effective after treatment with anti-CSF-1 antibodies (Paulus et al. 2006). Moreover, the mAb inhibiting the CSF-1R (RG7155) was proved both in vitro and in vivo to decrease F4/80^+^ TAMs accompanied by an increase in the CD8^+^/CD4^+^ T-cell ratio (Ries et al. 2014). Administration of RG7155 to patients led to striking reductions of CSF-1R^+^CD163^+^ macrophages in tumor tissues, which was manifested in objective clinical responses in diffuse-type giant cell tumor patients (Ries et al. 2014). Furthermore, the efficiency of the humanized antibody specific for M-CSF (MCS110) is under clinical investigation of a phase 2 study (clinical trials NCT02435680 and NCT01643850).

In several studies, the use of M-CSF receptor inhibitors decreased the number of TAMs in tumor tissue, suppressed tumor growth, and significantly decreased angiogenesis and metastasis. Oral administration of JNJ-28312141, an FMS-related receptor tyrosine kinase-3 (FLT3) inhibitor, caused dose-dependent suppression of human non-small cell lung carcinoma growth and reduced tumor vasculature in nude mice, which correlated with marked reductions in F4/80^+^ TAMs (Manthey et al. 2009). Another factor, PLX3397, was developed as a selective FLT3 inhibitor for hematological malignancies, but it functions as an inhibitor of CSF-1 receptor-associated kinases. In several works, the use of PLX3397 caused a decrease in tumor growth in the case of, e.g., glioblastoma (Butowski et al. 2016; Stafford et al. 2016), melanoma (Ngiow et al. 2015; Sluijter et al. 2014), and gastrointestinal stromal tumor (Kim et al. 2014). There are several phase 1/2 clinical trials that examine the efficiency of PLX397 in the treatment of different tumors (clinical trials NCT02584647, NCT02071940, NCT02472275, NCT02452424, NCT02371369, NCT01596751, NCT02401815, NCT01349049, NCT02734433, NCT01790503, NCT01525602, NCT01042379, and NCT02777710). Another CSF-1R inhibitor, BLZ945, was shown in preclinical studies as a potent antitumor drug in glioblastoma, breast carcinoma, and cervical carcinoma models. It was detected as a strong factor converting tumorigenic macrophages with the M2 phenotype into the antitumor M1 macrophages (Pyonteck et al. 2013; Strachan et al. 2013). Moreover, the efficiency of BLZ945 as a single agent or BLZ945 in combination with other factors in advanced solid tumors is under clinical investigation of phase 1/2 study (clinical trial NCT02829723). Another CSF-1 inhibitor (ARRY-382) is also under investigation in patients with selected advanced or metastatic cancers in a phase 1 study (clinical trial NCT01316822). The blockade of CSF-1 signaling by means of a mAb is also under investigation. Currently, two open clinical trials are recruiting participants. The first study examines the immunomodulatory activity of an anti-CSF-1R antibody (LY3022855, IMC-CS4) in patients with advanced breast or prostate cancer (clinical trial NCT02265536). The other study will evaluate the safety and activity of an anti-CSF-1R antibody (pexidartinib) in patients with metastatic/advanced pancreatic or colorectal cancers combined with an anti-programmed cell death ligand 1 antibody (clinical trial NCT02777710).

The CXCR-4 is another receptor involved in the growth and metastasis of cancer cells. Recent studies have proved that the CXCR-4 and its ligand CXCL-12 (SDF-1) are promising targets for cancer therapy (Domanska et al. 2013; Wang et al. 2016). Numerous clinical studies proved the effectiveness of plerixafor (AMD3100), a CXCR-4 inhibitor as well as CTCE-9908, a CXCL-12 peptide analogue, in cancer treatment (Domanska et al. 2013). As macrophages express CXCR-4, this allows them to migrate along the gradient of CXCL-12. It was shown that CXCL-12 production by tumor cells in mice breast cancer model results in increased macrophage number at the tumor side (Boimel et al. 2012). Moreover, CXCL-12 produced by the multiple myeloma cells induced monocyte migration and the use of anti-CXCR-4 antibodies significantly abrogated monocyte recruitment (Beider et al. 2014). In mice, pharmacological blocking of CXCR-4 by plerixafor (AMD3100) blocked the post-sepsis-induced melanoma progression, accumulation of TAMs, and TAMs in situ proliferation (Mota et al. 2016). Furthermore, the pharmacologic blockade of CXCR-4 by plerixafor in mice selectively reduced the number of M2-related TAMs and tumor revascularization and regrowth (Hughes et al. 2015).

## Decreasing the population of TAMs

Another approach to treat tumors is to decrease the population of TAMs. There are two approaches; one is to reduce the population of monocytes in the blood, while the other is to reduce the number of macrophages already existing in tumor tissue. Certain anticancer treatments have been reported to elicit therapeutic responses by manipulating the number of macrophages in tumor tissues.

Bisphosphonates, i.e., inorganic analogues of pyrophosphonate, are potent inhibitors of osteoclast-mediated bone resorption; however, there is a growing body of evidence supporting their antitumor activity, most probably by influencing TAMs (Rogers and Holen 2011). Many studies have shown that bisphosphonates usually packed in liposomes induce macrophage apoptosis in vitro and in vivo (Van Acker et al. 2016). Moreover, they were proved to inhibit the release of proangiogenic factors and affect tumor macrophages in vivo by reversing their polarity to the protumoral phenotype. Clodronate-loaded liposomes (clondlip) were proved to decrease the numbers of monocytes and macrophages, which correlated with a decrease in tumor growth in different animal models. Reduction of blood monocytes by subcutaneous administration of liposomal clodronate markedly reduced the number of bone and muscle metastasis in a mouse lung cancer model (Hiraoka et al. 2008). Moreover, depletion of TAMs by clodronate-loaded liposomes augmented the inhibitory effect of sorafenib on tumor angiogenesis, growth, and metastasis in a hepatocellular carcinoma (HCC) xenograft model (Zhang et al. 2010). Clodronate-liposome treatment in murine F9 teratocarcinoma and human A673 Ewing’s sarcoma tumor models efficiently depleted TAMs and significantly inhibited tumor growth (Zeisberger et al. 2006). Recently, an antimacrophage activity of liposomal clodronate has been confirmed in a hepatocarcinoma mouse model (Piaggio et al. 2016). Moreover, depletion of TAMs with clodronate-loaded liposomes increased the antiangiogenic and antitumor effects of anti-VEGF antibodies in subcutaneous tumor models (Priceman et al. 2010).

Trabectedin (ET743, Yondelis) is a natural alkaloid derived initially from the Caribbean tunicate, which was proved to have strong anticancer properties. Based on the favorable results of numerous phases 1, 2, and 3 clinical trials, trabectedin gained full marketing approval for treatment of ovarian cancer and soft-tissue sarcomas from the European Commission in 2015 and gained the United States Food and Drug Administration (US FDA) approval for unresectable or metastatic liposarcoma or leiomyosarcoma in the same year (Gordon et al. 2016). Furthermore, the efficiency of trabectedin in treatment of many solid tumors alone or in combination with other drugs is under numerous clinical investigations of phases 1, 2, and 3 studies. Preclinical studies showed that trabectedin strongly inhibited tumor growth by inducing double-strand breaks in DNA and interrupting the cell cycle (Gordon et al. 2016). However, trabectedin does not only affect tumor cells. There are numerous data showing a strong influence of trabectedin on monocytes and TAMs. Trabectedin was proved to decrease the number of TAMs in tumor tissue by inducing apoptosis of monocytes and macrophages (Allavena et al. 2005). Trabectedin activates caspase 8 and induces apoptosis with involvement of TRAIL-R2, a death receptor present exclusively on TAMs (Germano et al. 2013; Kodumudi et al. 2010). Moreover, it decreases the production of several protumoral cytokines and chemokines such as CCL-2, CXCL-8, IL-6, and VEGF produced by TAMs (Allavena et al. 2005; Germano et al. 2010). The importance of trabectedin influence on TAMs in treatment of solid tumors was proved by Germano et al. (2013) in an experiment on mice implanted with trabectedin-resistant tumor cells. However, when applied in vivo, trabectedin strongly decreased tumor growth and the number of TAMs in tumor tissue.

Another anticancer strategy is to deplete TAMs by inducing cytotoxic CD8^+^T cells, which can recognize TAMs as a target and specifically kill them. There are several proteins specific for TAMs; one of them, legumain, a lysosomal endopeptidase, was shown to serve as a target for cancer therapy (Liu et al. 2003). A legumain-expressing DNA vaccine was proved to strongly reduce the number of TAMs, which resulted in marked suppression of tumor growth, metastasis, and angiogenesis in mouse models of metastatic breast cancers (Lewēn et al. 2008) and lung carcinoma (Xiang et al. 2008). There are a few modifications of the legumain DNA vaccine, for example, improvement of the delivery system, where alginic acid-coated chitosan nanoparticles were used as a carrier instead of *Salmonella thyphimurium* (Liu et al. 2013) or modification of the legumain protein to enhance the efficiency of DNA immunization (Smahel et al. 2014).

Depletion of TAMs by targeting their surface molecules is another approach in the cancer immunotherapy. A preclinical study showed that depletion of immune cells by alemtuzumab, an anti-CD52 antibody, in a murine ovarian cancer model caused a decrease in tumor growth and angiogenesis (Pulaski et al. 2009). Moreover, the efficiency of alemtuzumab in treatment of kidney, ovarian, and peritoneal tumors is under phase 1 clinical investigation (clinical trials NCT00637390, NCT00073879).

Scavenger receptor-A (SR-A) is expressed on TAMs and other immune cells (for example, dendritic cells) present in tumor tissue, which renders it as a target for immunotherapy. Moreover, in vivo studies showed that deficiency of SR-A inhibited tumor cell migration, progression, and metastasis (Neyen et al. 2013). Preclinical studies showed that administration of anti-SR-A immunotoxin substantially inhibited peritoneal tumor burden and ascites accumulation.

Another marker, the folate receptor β (FR-β), was proved to be exclusively expressed on macrophages present in tumor tissue (O’Shannessy et al. 2015; Puig-Kröger et al. 2009). Moreover, the expression of FR-β was found to be positively correlated with both the stage of cancer and the presence of lymph node metastases (Shen et al. 2015). Based on these data, it is believed that FR-β may constitute a good target for specific immunotherapy or delivery of therapeutic agents to TAMs (Kurahara et al. 2012). In a glioma model, depletion of TAMs by means of recombinant immunotoxin consisting of the FR-β mAb significantly reduced tumor growth (Nagai et al. 2009).

CD11b is a molecule present on bone-marrow-derived immune cells. Recent studies have recommended this molecule as an important oncogene and highlighted the potential of CD11b as a therapeutic target in colorectal cancer (Zhang et al. 2015). Moreover, an advantage of the anti-CD11b mAb treatment is that it can target both immunosuppressive macrophages and myeloid-derived suppressive cells (MDSC). Antibody-mediated depletion of CD11b myeloid cells reduced tumor regrowth after therapy in a murine model of head and neck tumors (Ahn et al. 2010) as well as in murine epithelial ovarian cancer (Khan et al. 2015).

The macrophage mannose receptor (CD206) is a marker abundantly expressed on M2 macrophages. Since TAMs are considered to be M2-polarized macrophages, the CD206 marker has recently been suggested as a desirable target for drug delivery into macrophages. Moreover, several studies have shown that CD206 expression is characteristic to TAMs present in many different tumors and correlates with the disease stage and progression (Ding et al. 2014; Laoui et al. 2011). Zhan et al. (2014) conjugated a polysaccharide from *Bletilla striata* with alendronate (4-amino-1-hydroxybutylidene 1.1-biphosphonate) for target delivery of alendronate to TAMs. Such a complex effectively eliminated TAMs, inhibited angiogenesis, and suppressed tumor progression in vivo in a mouse model of sarcoma (Zhan et al. 2014).

Moreover, Ngambenjawong et al. (2016) have identified an M2 macrophage-targeting peptide (M2pep) that binds preferentially to murine M2 macrophages and M2-like TAMs. A fusion peptide of M2pep with proapoptotic peptide KLA was further used to reduce a population of TAMs in vivo, but high concentrations and frequent administration were required due to the low binding affinity of M2pep for M2 macrophages. The goal of this study was to develop more potent constructs for depletion of TAMs by increasing the valency of both the M2pep targeting and KLA drug domains (Ngambenjawong et al. 2016).

## Transformation of TAMs into M1 Proinflammatory Macrophages

Manipulation of the phenotype of TAMs is a novel potential therapeutic approach to engage antitumor immunity. Several studies have proved that the phenotype of macrophages demonstrates a high level of plasticity and can be easily modulated by the external microenvironment. This gives an opportunity to target the suppressive TAMs and repolarize them into proinflammatory cells that will efficiently fight with tumor and activate other immune cells. There are several studies showing that activation of TLRs by means of lipopolysaccharide, CpG, and siRNA can activate TAMs into M1 macrophages. In addition, induction of the nuclear factor (NF)-κB or STAT3 pathways might result in transition of TAMs into M1 macrophages.

CD40, a receptor of the TNF-α family, is widely expressed by antigen-presenting cells (APC) including B cells, dendritic cells, and macrophages. Antibody binding to CD40-activated APC cells and, in consequence, triggered tumor specific T-cell immune response. Moreover, CD40-activated TAMs also exerted a direct tumoricidal effect (Vonderheide et al. 2013b). The anti-CD40 antibody was proved to activate tumor TAMs and block tumor growth in mice and human models of pancreatic carcinoma (Beatty et al. 2011), in a mice glioma model (Kosaka et al. 2014), and in a mice model of melanoma (Alderson et al. 2013). There are three most extensively tested anti-CD40 antibodies (Vonderheide and Glennie 2013). CP-870,893 (Pfizer/VLST), a strong agonist of CD40, is a fully human IgG2 with well-proved antitumor immunity in patients with solid tumors, including melanoma and pancreatic cancer (Beatty et al. 2013; Vonderheide et al. 2007, 2013b). ChiLob 7/4 is an intermediate CD40 agonist and chimeric IgG1, which was also proved to induce the proinflammatory cytokine and gave promising results in phase 1 clinical studies in CD40-expressing solid tumors and diffuse large B-cell lymphoma resistant to conventional therapy (Chowdhury et al. 2014; Johnson et al. 2015). Dacetuzumab is a humanized IgG1 anti-CD40 antibody considered as a weak agonist of CD40; several clinical trials have shown its efficiency in treatment of hematological malignancies (Vonderheide et al. 2013a).

There are several factors able to activate the immune response and TAMs. Thymosin-α, a thymus hormone, was proved to activate the immune system by several mechanisms, including stimulation of T-cell differentiation, activation of natural killer, dendritic cells, and macrophages (Garaci et al. 2012). There are studies showing that delivery of thymosin-α significantly activates TAMs and switches them toward proinflammatory subsets producing IL-1, TNF-α, ROS, and NO. In turn, they delay tumor growth, and prolong survival in mice with Dalton lymphoma (Shrivastava et al. 2004, 2005). Moreover, several clinical trials have confirmed that thymosin-α 1 prolonged survival in patients with metastatic melanoma and advanced non-small cell lung cancer (Garaci et al. 2012).

Several studies demonstrate that bacteria can also serve as a factor influencing TAMs. It was shown that attenuated *Listeria monocytogenes* used in treatment of ovarian cancer in mice, targeted immunosuppressive TAMs in the cancer microenvironment, and repolarized these cells to the proinflammatory phenotype (Le et al. 2012; Lizotte et al. 2014). Moreover, injection of attenuated *Shigella flexneri* into breast cancer bearing mice resulted in apoptosis of TAMs followed by a 74% reduction in tumors (Galmbacher et al. 2010).

β-Glucan, a yeast-derived polysaccharide, is a potent immunomodulator that was shown to possess anticancer properties (Albeituni et al. 2016). β-Glucan was shown to convert immunosuppressive TAMs into the M1 macrophages (Liu et al. 2015) and causes significant loss of the suppressive function of monocytic MDSC (Tian et al. 2013); in both cases, the β-glucan treatment significantly delayed tumor progression. The use of β-glucan was checked in a phase 1 clinical trial of neuroblastoma treatment; however, the outcome was uncertain because of the complexity and heterogeneity of prior patient therapies (Kushner et al. 2014). In another study, a β-glucan polymer (PPG) showed compelling but modest activity in a phase 2 clinical multicancer study (Segal et al. 2016). Furthermore, the efficiency of β-glucan is currently under clinical investigation of phase 1 and 2 studies (clinical trials NCT00911560, NCT00492167).

Sorafenib (Nexavar) is a well-known anticancer drug approved for use in HCC (Llovet et al. 2008). Recent studies have shown that this oral multikinase inhibitor inhibited macrophage-induced epithelial–mesenchymal transition in human HCC and their migration. It also reduced macrophage infiltration in tumors (Deng et al. 2016).

Metformin is a well-known antidiabetic drugs, which has recently been proved by several clinical trials to have a positive effect in treatment of many carcinomas (Sośnicki et al. 2016). However, the mechanisms of metformin action to inhibit tumor growth and metastasis are not fully understood. The latest data have shown that metformin may inhibit M2-like polarization of macrophages both in vitro and in vivo and greatly reduce the number of metastases in a murine model of Lewis lung carcinoma (Ding et al. 2015).

Embelin is a small-molecule inhibitor of the X-linked inhibitor of apoptosis protein, which also influences the NF-κB and STAT3 pathways. In colitis-associated cancer model, embelin significantly decreased the production of key proinflammatory cytokines and reduced polarization of M2 macrophages (Wu et al. 2016). Moreover, embelin was shown to inhibit growth of colon and pancreatic tumors, but the mechanisms of its action are not known (Dai et al. 2009; Peng et al. 2014).

Moreover, it has recently been found that the pattern recognizing scavenger receptor MARCO, characteristic for suppressive TAMs, is linked to the clinical outcome of many tumors. A monoclonal antibody against this receptor induced antitumor activity in breast and colon cancer and melanoma models through reprogramming the population of TAMs to a proinflammatory phenotype. This antitumor activity was dependent on the inhibitory activity of the Fc-receptor and FcγRIIB (Georgoudaki et al. 2016).

The use of siRNA in the treatment of cancer has been intensively investigated (Resnier et al. 2013) and several anticancer siRNA-based drugs have already entered clinical trials (Lee et al. 2016). Moreover, there are a few trials to use siRNA to reprogram TAMs and transform them into M1 macrophages. An in vitro study showed that inhibition of the NF-κB activity by siRNA caused a significant decrease in expression of IL-10, VEGF, and MMP-9 and a significant increase in Th1 cytokine production in mouse peritoneal M2 macrophages (Kono et al. 2014). Ortega et al. (2015) reported that mannosylated polymer nanoparticles were able to deliver siRNA to TAMs in vitro and in vivo in the peritoneum of ovarian tumor bearing mice and in TAMs present in lung metastases. In addition, delivery of siRNA targeting the NF-κB pathway into TAMs of tumor bearing mice resulted in induction of a proinflammatory immunogenic phenotype in transfected macrophages and yielded an antitumor phenotype (Ortega et al. 2016). Moreover, dual targeted immunotherapy via in vivo delivery of biohybrid siRNA-peptide nanoparticles to tumor macrophages substantially reduced the recruitment of TAMs in lung tumor tissue, reduced tumor size, and increased animal survival (Conde et al. 2015).

MicroRNAs (miRNAs) are another class of small non-coding RNA molecules that can regulate the expression of proteins at the post-transcriptional level. Recent studies have revealed an important role of miRNA in development and activation of macrophages (Squadrito et al. 2013; Wei and Schober 2016). Recent studies have shown that overexpression of miR-155 can reprogram anti-inflammatory, protumoral TAMs to proinflammatory, and antitumor M1 macrophages (Cai et al. 2012; Su et al. 2016). Moreover, the enforced expression of miR-511-3p turned down the protumoral genes of TAMs and inhibited tumor growth (Squadrito et al. 2012). In addition, increased expression of miR-26a in a mouse model of HCC suppressed tumor growth, M-CSF expression, and infiltration of macrophages in tumors (Chai et al. 2015). The expression of miR-26a was inversely correlated with M-CSF expression and macrophage infiltration in tumor tissues from patients with HCC (Chai et al. 2015).

CpG oligodeoxynucleotides (CpG ODN), i.e., short single-stranded synthetic DNA molecules, are recognized by TLR-9 and act as a strong immunostimulant. Several preclinical and clinical studies have proved their role as a vaccine adjuvant to treat various tumors (Shirota et al. 2015). Several studies have shown that intratumor injection of CpG ODN reduces the number and suppressive activity of tumor-infiltrating monocyte-derived suppressor cells and induces their maturation into M1 macrophages (Shirota et al. 2012; Shirota and Klinman 2012). Moreover, several synthetic TLR-9 agonists have been developed for clinical grade use and displayed substantial efficacy in preclinical and clinical models (Holtick et al. 2011). Recent studies have demonstrated that the TLR-9 agonist effectively reduces the number of TAMs, MDSC, and regulatory T cells in a mouse cervical cancer model (Chang et al. 2014). Moreover, Le Noci et al. (2015) observed that CpG reduced the presence of M2 suppressive macrophages in the lungs of B16 melanoma-bearing mice. Another method that can modulate macrophage polarization was described by Huang et al. 2012. Anti-IL-10 CpG ODN and anti-10RA antisense oligonucleotides were used in combination and such a complex, specifically in an allograft hepatoma murine model, suppressed the protumor function and stimulated the antitumor activities of TAMs (Huang et al. 2012). Furthermore, the synergy in the anti-CD40, CpG, and monophosphoryl lipid A (MPL) treatment was observed to activate TAMs via CD40/TLR9 ligation to kill tumor cells in vitro and inhibit tumor growth in vivo (Buhtoiarov et al. 2006; Shi et al. 2015). Another approach uses gold nanoparticles containing modified CpG ODN as an immunostimulant of innate immunity. These complexes increased the macrophage and dendritic cell stimulatory effect significantly inhibited B16 melanoma tumor growth in mice, and promoted survival in mice, compared to treatments with free CpG (Lin et al. 2013).

Polarization of M2 macrophages to the M1 macrophages was also observed under the influence of host-produced histidine-rich glycoprotein (HRG). It was proved that HRG inhibits tumor growth and metastasis, promotes antitumor immune responses, and induces vessel normalization (Johnson et al. 2014). HRG reversed polarization of TAMs into tumor-inhibiting M1 macrophages via downregulation of the placental growth factor, a member of the VEGF family (Rolny et al. 2011). In addition, a phase 3 trial (NCT01664169) identified HRG as a weakly positive prognostic biomarker (Roberts et al. 2012).

Moreover, there are attempts to modulate macrophage plasticity by regulating the activation of core cell signaling pathways affecting macrophage differentiation. The transcription factor NF-κB is a key factor in cancer-related inflammation and is critical for macrophage activation in response to inflammatory stimuli (D’Ignazio et al. 2016). The activation of NF-κB is crucial for the activation of macrophages and induction of expression of many proinflammatory cytokines typical for the M1 macrophages (Hagemann et al. 2009). On the other hand, other reports indicate that the activation of NF-κB is also required for tumor development in many cancer models, due to the release of proinflammatory cytokines such as TNF-α and IL-6, which can trigger prosurvival signals for tumor cells supporting their growth and progression (Karin and Greten 2005). Hagemann et al. (2008) showed that inhibition of NF-κB signaling in TAMs switched them into M1 macrophages, which is cytotoxic to tumor cells, and promoted regression of advanced tumors in vivo. However, according to other studies, activation of NF-κB activates TAMs and induces their tumor cytotoxicity. It was shown that the activation of NF-κB in macrophages leads to increased production of inflammatory cytokines and reduction of lung metastasis in a mouse breast cancer model (Connelly et al. 2011). Moreover, continuous IKKβ signaling in myeloid-lineage cells exhibited enhanced antitumor immunity and reduced tumor outgrowth in a melanoma tumor model (Yang et al. 2014). Activation of NF-κB in TAMs by means of siRNA delivering nanoparticles switched them into proinflammatory cells (Ortega et al. 2016).

Differentiation of TAMs from monocytes is controlled by other signaling pathways as well. It has been shown that the transcription factor STAT3 is consistently active in many tumors and acts as a negative regulator of macrophage activation (Lang 2005). It was shown that, by blocking the STAT3 activity, the phenotype of macrophages could be switched into proinflammatory with high expression of IL-12 and RANTES (Cheng et al. 2003). Several known STAT3 inhibitors have been proved to influence the phenotype of TAMs and have an influence on treatment of tumors. It was shown that upregulation of CD45 tyrosine phosphatase activity in myeloid cells exposed to hypoxia downregulated STAT3. This effect was mediated by disruption of CD45 protein dimerization regulated by sialic acid and affected macrophage differentiation (Kumar et al. 2016). The STAT3 transcription factor was the target in the experiments of Fujiwara et al. (2014). Among the 200 purified natural products examined, two triterpenoid compounds, i.e., corosolic acid and oleanolic acid, inhibited macrophage polarization to the M2 phenotype by suppressing STAT3 activation. Moreover, both substances directly inhibited tumor growth and sensitized tumors to anticancer drugs. In vivo, corosolic acid significantly suppressed subcutaneous tumor development and lung metastasis in a murine sarcoma model (Fujiwara et al. 2014). Moreover, it has been proved that tyrosine kinase inhibitors can inhibit STAT3 and influence macrophage differentiation. Sunitinib has shown its inhibitory effect on STAT3 in macrophages (Xin et al. 2009). In addition, sorafenib was proved to reverse TAMs to protumoral cells (Edwards and Emens 2010). Sunitinib is currently considered the standard of care for the first-line treatment of metastatic clear cell renal cell carcinoma (Minardi et al. 2015). Moreover, several clinical trials have checked its efficiency in treatment of small lung carcinoma (Abdelraouf et al. 2016), prostate cancer (Armstrong et al. 2016), and thymic malignancies (Remon et al. 2016), whereas sorafenib has been approved for the treatment of primary advanced renal cell carcinoma and advanced primary liver cancer (Pitoia and Jerkovich 2016). Moreover, many other small-molecule tyrosine kinase inhibitors, including vandetanib, cabozantinib, and lenvatinib, are now FDA-approved drugs for thyroid cancer and have shown clinical benefits in advanced thyroid cancer (Cabanillas and Habra 2016). To date, the US FDA has approved 28 small-molecule kinase inhibitors, half of which were approved in the past 3 years (Wu et al. 2015).

## TAMs as Carriers of Anticancer Drugs

The development of nanocarriers for treatment of neurodegenerative diseases or for application of antiretroviral therapy against HIV prompted scientists to apply nanocarriers in antitumor therapy. It has been detected that gold nanoshells can be actively internalized by macrophages and delivered into hypoxic regions of tumors (Choi et al. 2007). In vitro experiments have shown that gold nanoshells accumulated in cancer spheroids can cause cell death by photothermal treatment. Gold nanorods with a macrolide motif were also demonstrated to accumulate in macrophages present in the tumor and caused cancer cell death around these macrophages. This type of ablation therapy is one of the most promising strategies of cancer treatment. Prednisone phosphate encapsulated in long-circulating liposomes (LCL-PLP) exerts antitumor activity through the inhibition of tumor angiogenesis. One of the major inhibitory actions of LCL-PLP on tumor growth is reduction of proangiogenic factors produced by TAMs (Banciu et al. 2008). Another nanotechnology was applied in construction of an anticancer agent by Wang et al. (2012). A magnetic shell from iron oxide/iron was combined with topoisomerase I inhibitor SN38 via a carboxyl esterase linker and loaded into RAW 264.7 macrophages. When they were delivered into the tumor site, magnetic hyperthermia released SN38 and exerted an anticancer effect (Wang et al. 2012). Similar macrophage-based combination therapy was designed by Ikehara et al. (2006). They constructed magnetic nanoparticles coated with mannose and loaded with 5-fluorouracil. When injected into the peritoneal cavity of mice with breast carcinoma, the particles were internalized by peritoneal macrophages. Controlled release of 5-fluorouracil and tumor growth inhibition was observed when an electromagnetic field was applied in a mouse intraperitoneal metastatic model.

Tumor-infiltrating macrophages may also be vehicles for anti-inflammatory and antitumor cytokines. Escobar et al. (2014) described efforts in which the human gene coding interferon (IFN)-α was introduced into myeloid progeny mouse cells. Mice chimeric for the IFN-α-expressing macrophages showed activation of innate and adaptive immunity against breast cancer and inhibited disease progression (Escobar et al. 2014).

## Conclusion

The increased number of clinical and preclinical studies proved the importance of choosing TAMs as a target of anticancer therapies. Multiple approaches are undertaken to decrease the number of suppressive macrophages in the tumor tissue. Most probably, inhibition of recruitment of monocytes into the tumor site, specific killing suppressive M2 macrophages, or modification of immunosuppressive TAMs into the antitumor phenotype could be efficiently used in combined antitumor immunotherapies in future treatment. Moreover, it is important to focus on development of drugs that could specifically target TAMs, which could increase the specificity of the treatment and reduce their toxicity to other cells in the organism.

## References

[CR1] Abdelraouf F, Smit E, Hasan B (2016). Sunitinib (SU11248) in patients with chemo naive extensive small cell lung cancer or who have a “chemosensitive” relapse: a single-arm phase II study (EORTC-08061). Eur J Cancer.

[CR2] Ahn GO, Tseng D, Liao CH (2010). Inhibition of Mac-1 (CD11b/CD18) enhances tumor response to radiation by reducing myeloid cell recruitment. Proc Natl Acad Sci USA.

[CR3] Albeituni SH, Ding C, Liu M (2016). Yeast-derived particulate β-glucan treatment subverts the suppression of myeloid-derived suppressor cells (MDSC) by inducing polymorphonuclear MDSC apoptosis and monocytic MDSC differentiation to APC in cancer. J Immunol.

[CR4] Alderson KL, Luangrath M, Elsenheimer MM (2013). Enhancement of the anti-melanoma response of Hu14.18K322A by αCD40 + CpG. Cancer Immunol Immunother.

[CR5] Allavena P, Signorelli M, Chieppa M (2005). Anti-inflammatory properties of the novel antitumor agent yondelis (trabectedin): inhibition of macrophage differentiation and cytokine production. Cancer Res.

[CR6] Allavena P, Sica A, Solinas G (2008). The inflammatory micro-environment in tumor progression: the role of tumor-associated macrophages. Crit Rev Oncol Hematol.

[CR7] Angevin E, Tabernero J, Elez E (2014). A phase I/II, multiple-dose, dose-escalation study of siltuximab, an anti-interleukin-6 monoclonal antibody, in patients with advanced solid tumors. Clin Cancer Res.

[CR8] Armstrong AJ, Halabi S, Healy P (2016). A phase 2 multimodality trial of docetaxel/prednisone with sunitinib followed by salvage radiation therapy in men with PSA recurrent prostate cancer after radical prostatectomy. Prostate Cancer Prostatic Dis.

[CR9] Banciu M, Metselaar JM, Schiffelers RM (2008). Liposomal glucocorticoids as tumor-targeted anti-angiogenic nanomedicine in B16 melanoma-bearing mice. J Steroid Biochem Mol Biol.

[CR10] Beatty GL, Chiorean EG, Fishman MP (2011). CD40 agonists alter tumor stroma and show efficacy against pancreatic carcinoma in mice and humans. Science.

[CR11] Beatty GL, Torigian DA, Chiorean EG (2013). A phase I study of an agonist CD40 monoclonal antibody (CP-870,893) in combination with gemcitabine in patients with advanced pancreatic ductal adenocarcinoma. Clin Cancer Res.

[CR12] Beider K, Bitner H, Leiba M (2014). Multiple myeloma cells recruit tumor-supportive macrophages through the CXCR4/CXCL12 axis and promote their polarization toward the M2 phenotype. Oncotarget.

[CR13] Biragyn A, Longo DL (2012). Neoplastic “Black Ops”: cancer’s subversive tactics in overcoming host defenses. Semin Cancer Biol.

[CR14] Bissell MJ, Hines WC (2011). Why don’t we get more cancer? A proposed role of the microenvironment in restraining cancer progression. Nat Med.

[CR15] Boimel PJ, Smirnova T, Zhou ZN (2012). Contribution of CXCL12 secretion to invasion of breast cancer cells. Breast Cancer Res.

[CR16] Brana I, Calles A, LoRusso PM (2015). Carlumab, an anti-C-C chemokine ligand 2 monoclonal antibody, in combination with four chemotherapy regimens for the treatment of patients with solid tumors: an open-label, multicenter phase 1b study. Target Oncol.

[CR17] Bruchard M, Mignot G, Derangère V (2013). Chemotherapy-triggered cathepsin B release in myeloid-derived suppressor cells activates the Nlrp3 inflammasome and promotes tumor growth. Nat Med.

[CR18] Buhtoiarov IN, Lum HD, Berke G (2006). Synergistic activation of macrophages via CD40 and TLR9 results in T cell independent antitumor effects. J Immunol.

[CR19] Butowski N, Colman H, De Groot JF (2016). Orally administered colony stimulating factor 1 receptor inhibitor PLX3397 in recurrent glioblastoma: an Ivy Foundation Early Phase Clinical Trials Consortium phase II study. Neuro Oncol.

[CR20] Cabanillas ME, Habra MA (2016). Lenvatinib: role in thyroid cancer and other solid tumors. Cancer Treat Rev.

[CR21] Cabarcas SM, Mathews LA, Farrar WL (2011). The cancer stem cell niche–there goes the neighborhood?. Int J Cancer.

[CR22] Cai X, Yin Y, Li N (2012). Re-polarization of tumor-associated macrophages to pro-inflammatory M1 macrophages by microRNA-155. J Mol Cell Biol.

[CR23] Chai ZT, Zhu XD, Ao JY (2015). microRNA-26a suppresses recruitment of macrophages by down-regulating macrophage colony-stimulating factor expression through the PI3K/Akt pathway in hepatocellular carcinoma. J Hematol Oncol.

[CR24] Chang LS, Leng CH, Yeh YC (2014). Toll-like receptor 9 agonist enhances anti-tumor immunity and inhibits tumor-associated immunosuppressive cells numbers in a mouse cervical cancer model following recombinant lipoprotein therapy. Mol Cancer.

[CR25] Chen R, Chen B (2015). Siltuximab (CNTO 328): a promising option for human malignancies. Drug Des Dev Ther.

[CR26] Cheng F, Wang HW, Cuenca A (2003). A critical role for Stat3 signaling in immune tolerance. Immunity.

[CR27] Choi MR, Stanton-Maxey KJ, Stanley JK (2007). A cellular Trojan Horse for delivery of therapeutic nanoparticles into tumors. Nano Lett.

[CR28] Chowdhury F, Johnson PW, Glennie MJ (2014). Ex vivo assays of dendritic cell activation and cytokine profiles as predictors of in vivo effects in an anti-human CD40 monoclonal antibody ChiLob 7/4 phase I trial. Cancer Immunol Res.

[CR29] Conde J, Bao C, Tan Y (2015). Dual targeted immunotherapy via in vivo delivery of biohybrid RNAi-peptide nanoparticles to tumour-associated macrophages and cancer cells. Adv Funct Mater.

[CR30] Connelly L, Barham W, Onishko HM (2011). NF-kappaB activation within macrophages leads to an anti-tumor phenotype in a mammary tumor lung metastasis model. Breast Cancer Res.

[CR31] Coward J, Kulbe H, Chakravarty P (2011). Interleukin-6 as a therapeutic target in human ovarian cancer. Clin Cancer Res.

[CR32] D’Ignazio L, Bandarra D, Rocha S (2016). NF-κB and HIF crosstalk in immune responses. FEBS J.

[CR33] Dai Y, Qiao L, Chan KW (2009). Peroxisome proliferator-activated receptor-gamma contributes to the inhibitory effects of Embelin on colon carcinogenesis. Cancer Res.

[CR34] De Palma M, Lewis CE (2013). Macrophage regulation of tumor responses to anticancer therapies. Cancer Cell.

[CR35] Deng YR, Liu WB, Lian ZX (2016). Sorafenib inhibits macrophage-mediated epithelial-mesenchymal transition in hepatocellular carcinoma. Oncotarget.

[CR36] Ding P, Wang W, Wang J (2014). Expression of tumor-associated macrophage in progression of human glioma. Cell Biochem Biophys.

[CR37] Ding L, Liang G, Yao Z (2015). Metformin prevents cancer metastasis by inhibiting M2-like polarization of tumor associated macrophages. Oncotarget.

[CR38] Dirkx AE, Oude Egbrink MG, Wagstaff J (2006). Monocyte/macrophage infiltration in tumors: modulators of angiogenesis. J Leukoc Biol.

[CR39] Domanska UM, Kruizinga RC, Nagengast WB (2013). A review on CXCR4/CXCL12 axis in oncology: no place to hide. Eur J Cancer.

[CR40] Edwards JP, Emens LA (2010). The multikinase inhibitor Sorafenib reverses the suppression of IL-12 and enhancement of IL-10 by PGE2 in murine macrophages. Int Immunopharmacol.

[CR41] Escobar G, Gentner B, Naldini L (2014). Engineered tumor-infiltrating macrophages as gene delivery vehicles for interferon-α activates immunity and inhibits breast cancer progression. Oncoimmunology.

[CR42] Fang WB, Yao M, Brummer G (2016). Targeted gene silencing of CCL2 inhibits triple negative breast cancer progression by blocking cancer stem cell renewal and M2 macrophage recruitment. Oncotarget.

[CR43] Fujiwara Y, Takeya M, Komohara Y (2014). A novel strategy for inducing the antitumor effects of triterpenoid compounds: blocking the protumoral functions of tumor-associated macrophages via STAT3 inhibition. Biomed Res Int.

[CR44] Galmbacher K, Heisig M, Hotz C (2010). Shigella mediated depletion of macrophages in a murine breast cancer model is associated with tumor regression. PLoS One.

[CR45] Garaci E, Pica F, Serafino A (2012). Thymosin α1 and cancer: action on immune effector and tumor target cells. Ann N Y Acad Sci.

[CR46] Georgoudaki AM, Prokopec KE, Boura VF (2016). Reprogramming tumor-associated macrophages by antibody targeting inhibits cancer progression and metastasis. Cell Rep.

[CR47] Germano G, Frapolli R, Simone M (2010). Antitumor and anti-inflammatory effects of trabectedin on human myxoid liposarcoma cells. Cancer Res.

[CR48] Germano G, Frapolli R, Belgiovine C (2013). Role of macrophage targeting in the antitumor activity of trabectedin. Cancer Cell.

[CR49] Gordon S, Martinez FO (2010). Alternative activation of macrophages: mechanism and functions. Immunity.

[CR50] Gordon EM, Sankhala KK, Chawla N (2016). Trabectedin for soft tissue sarcoma: current status and future perspectives. Adv Ther.

[CR51] Hagemann T, Lawrence T, McNeish I (2008). “Re-educating” tumor-associated macrophages by targeting NF-kappaB. J Exp Med.

[CR52] Hagemann T, Biswas SK, Lawrence T (2009). Regulation of macrophage function in tumors: the multifaceted role of NF-κB. Blood.

[CR53] Heusinkveld M, van der Burg SH (2011). Identification and manipulation of tumor associated macrophages in human cancers. J Transl Med.

[CR54] Hiraoka K, Zenmyo M, Watari K (2008). Inhibition of bone and muscle metastases of lung cancer cells by a decrease in the number of monocytes/macrophages. Cancer Sci.

[CR55] Hollmén M, Karaman S, Schwager S (2015). G-CSF regulates macrophage phenotype and associates with poor overall survival in human triple-negative breast cancer. Oncoimmunology.

[CR56] Holtick U, Scheulen ME, von Bergwelt-Baildon MS (2011). Toll-like receptor 9 agonists as cancer therapeutics. Expert Opin Investig Drugs.

[CR57] Huang Z, Zhang Z, Jiang Y (2012). Targeted delivery of oligonucleotides into tumor-associated macrophages for cancer immunotherapy. J Control Release.

[CR58] Hughes R, Qian B-Z, Rowan C (2015). Perivascular M2 macrophages stimulate tumor relapse after chemotherapy. Cancer Res.

[CR59] Ikehara Y, Niwa T, Biao L (2006). A carbohydrate recognition-based drug delivery and controlled release system using intraperitoneal macrophages as a cellular vehicle. Cancer Res.

[CR60] Jinushi M, Chiba S, Yoshiyama H (2011). Tumor-associated macrophages regulate tumorigenicity and anticancer drug responses of cancer stem/initiating cells. Proc Natl Acad Sci USA.

[CR61] Johnson LD, Goubran HA, Kotb RR (2014). Histidine rich glycoprotein and cancer: a multi-faceted relationship. Anticancer Res.

[CR62] Johnson P, Challis R, Chowdhury F (2015). Clinical and biological effects of an agonist anti-CD40 antibody: a Cancer Research UK phase I study. Clin Cancer Res.

[CR63] Karin M, Greten FR (2005). NF-kappaB: linking inflammation and immunity to cancer development and progression. Nat Rev Immunol.

[CR64] Karkera J, Steiner H, Li W (2011). The anti-interleukin-6 antibody siltuximab down-regulates genes implicated in tumorigenesis in prostate cancer patients from a phase I study. Prostate.

[CR65] Khan ANH, Kolomeyevskaya N, Singel KL (2015). Targeting myeloid cells in the tumor microenvironment enhances vaccine efficacy in murine epithelial ovarian cancer. Oncotarget.

[CR66] Kim TS, Cavnar MJ, Cohen NA (2014). Increased KIT inhibition enhances therapeutic efficacy in gastrointestinal stromal tumor. Clin Cancer Res.

[CR67] Kodumudi KN, Woan K, Gilvary DL (2010). A novel chemoimmunomodulating property of docetaxel: suppression of myeloid-derived suppressor cells in tumor bearers. Clin Cancer Res.

[CR68] Kono Y, Kawakami S, Higuchi Y (2014). In vitro evaluation of inhibitory effect of nuclear factor-kappaB activity by small interfering RNA on pro-tumor characteristics of M2-like macrophages. Biol Pharm Bull.

[CR69] Kosaka A, Ohkuri T, Okada H (2014). Combination of an agonistic anti-CD40 monoclonal antibody and the COX-2 inhibitor celecoxib induces anti-glioma effects by promotion of type-1 immunity in myeloid cells and T-cells. Cancer Immunol Immunother.

[CR70] Kubota Y, Takubo K, Shimizu T (2009). M-CSF inhibition selectively targets pathological angiogenesis and lymphangiogenesis. J Exp Med.

[CR71] Kumar V, Cheng P, Condamine T (2016). CD45 Phosphatase inhibits STAT3 transcription factor activity in myeloid cells and promotes tumor-associated macrophage differentiation. Immunity.

[CR72] Kurahara H, Takao S, Kuwahata T (2012). Clinical significance of folate receptor β-expressing tumor-associated macrophages in pancreatic cancer. Ann Surg Oncol.

[CR73] Kushner BH, Cheung IY, Modak S (2014). Phase I trial of a bivalent gangliosides vaccine in combination with β-glucan for high-risk neuroblastoma in second or later remission. Clin Cancer Res.

[CR74] Lang R (2005). Tuning of macrophage responses by Stat3-inducing cytokines: molecular mechanisms and consequences in infection. Immunobiology.

[CR75] Laoui D, Movahedi K, Van Overmeire E (2011). Tumor-associated macrophages in breast cancer: distinct subsets, distinct functions. Int J Dev Biol.

[CR76] Le Noci V, Tortoreto M, Gulino A (2015). Poly(I:C) and CpG-ODN combined aerosolization to treat lung metastases and counter the immunosuppressive microenvironment. Oncoimmunology.

[CR77] Le DT, Brockstedt DG, Nir-Paz R (2012). A live-attenuated Listeria vaccine (ANZ-100) and a live-attenuated Listeria vaccine expressing mesothelin (CRS-207) for advanced cancers: phase I studies of safety and immune induction. Clin Cancer Res.

[CR78] Lee SJ, Kim MJ, Roberts TM (2016). Delivery strategies and potential targets for siRNA in major cancer types. Adv Drug Deliv Rev.

[CR79] Lewēn S, Zhou H, Hu H (2008). A Legumain-based minigene vaccine targets the tumor stroma and suppresses breast cancer growth and angiogenesis. Cancer Immunol Immunother.

[CR80] Lim SY, Yuzhalin AE, Gordon-Weeks AN (2016). Targeting the CCL2-CCR2 signaling axis in cancer metastasis. Oncotarget.

[CR81] Lin AY, Almeida JPM, Bear A (2013). Gold nanoparticle delivery of modified CpG stimulates macrophages and inhibits tumor growth for enhanced immunotherapy. PLoS One.

[CR82] Liu C, Sun C, Huang H (2003). Overexpression of legumain in tumors is significant for invasion/metastasis and a candidate enzymatic target for prodrug therapy. Cancer Res.

[CR83] Liu Z, Lv D, Liu S (2013). Alginic acid-coated chitosan nanoparticles loaded with legumain DNA vaccine: effect against breast cancer in mice. PLoS One.

[CR84] Liu M, Luo F, Ding C (2015). Dectin-1 activation by a natural product β-glucan converts immunosuppressive macrophages into an M1-like phenotype. J Immunol.

[CR85] Lizotte PH, Baird JR, Stevens CA (2014). Attenuated *Listeria monocytogenes* reprograms M2-polarized tumor-associated macrophages in ovarian cancer leading to iNOS-mediated tumor cell lysis. Oncoimmunology.

[CR86] Llovet JM, Ricci S, Mazzaferro V (2008). Sorafenib in advanced hepatocellular carcinoma. N Engl J Med.

[CR87] Loberg RD, Ying C, Craig M (2007). Targeting CCL2 with systemic delivery of neutralizing antibodies induces prostate cancer tumor regression in vivo. Cancer Res.

[CR88] Manthey CL, Johnson DL, Illig CR (2009). JNJ-28312141, a novel orally active colony-stimulating factor-1 receptor/FMS-related receptor tyrosine kinase-3 receptor tyrosine kinase inhibitor with potential utility in solid tumors, bone metastases, and acute myeloid leukemia. Mol Cancer Ther.

[CR89] Mantovani A, Sica A (2010). Macrophages, innate immunity and cancer: balance, tolerance, and diversity. Curr Opin Immunol.

[CR90] Minardi D, Quaresima L, Santoni M (2015). Recent aspects of sunitinib therapy in patients with metastatic clear-cell renal cell carcinoma: a systematic review of the literature. Curr Urol Rep.

[CR91] Mitchem JB, Brennan DJ, Knolhoff BL (2013). Targeting tumor-infiltrating macrophages decreases tumor-initiating cells, relieves immunosuppression, and improves chemotherapeutic responses. Cancer Res.

[CR92] Moisan F, Francisco EB, Brozovic A (2014). Enhancement of paclitaxel and carboplatin therapies by CCL2 blockade in ovarian cancers. Mol Oncol.

[CR93] Mota JM, Leite CA, Souza LE (2016). Post-sepsis state induces tumor-associated macrophage accumulation through CXCR4/CXCL12 and favors tumor progression in mice. Cancer Immunol Res.

[CR94] Moughon DL, He H, Schokrpur S (2015). Macrophage blockade using CSF1R inhibitors reverses the vascular leakage underlying malignant ascites in late-stage epithelial ovarian cancer. Cancer Res.

[CR95] Murdoch C, Muthana M, Coffelt SB, Lewis CE (2008). The role of myeloid cells in the promotion of tumour angiogenesis. Nat Rev Cancer.

[CR96] Nagai T, Tanaka M, Tsuneyoshi Y (2009). Targeting tumor-associated macrophages in an experimental glioma model with a recombinant immunotoxin to folate receptor beta. Cancer Immunol Immunother.

[CR97] Neyen C, Plüddemann A, Mukhopadhyay S (2013). Macrophage scavenger receptor a promotes tumor progression in murine models of ovarian and pancreatic cancer. J Immunol.

[CR98] Ngambenjawong C, Cieslewicz M, Schellinger JG (2016). Synthesis and evaluation of multivalent M2pep peptides for targeting alternatively activated M2 macrophages. J Control Release.

[CR99] Ngiow SF, Meeth KM, Stannard K (2015). Co-inhibition of colony stimulating factor-1 receptor and BRAF oncogene in mouse models of BRAF(V600E) melanoma. Oncoimmunology.

[CR100] Noy R, Pollard JW (2014). Tumor-associated macrophages: from mechanisms to therapy. Immunity.

[CR101] Nywening TM, Wang-Gillam A, Sanford DE (2016). Targeting tumour-associated macrophages with CCR2 inhibition in combination with FOLFIRINOX in patients with borderline resectable and locally advanced pancreatic cancer: a single-centre, open-label, dose-finding, non-randomised, phase 1b trial. Lancet Oncol.

[CR102] O’Shannessy DJ, Somers EB, Wang LC (2015). Expression of folate receptors alpha and beta in normal and cancerous gynecologic tissues: correlation of expression of the beta isoform with macrophage markers. J Ovarian Res.

[CR103] Ortega RA, Barham WJ, Kumar B (2015). Biocompatible mannosylated endosomal-escape nanoparticles enhance selective delivery of short nucleotide sequences to tumor associated macrophages. Nanoscale.

[CR104] Ortega RA, Barham W, Sharman K (2016). Manipulating the NF-κB pathway in macrophages using mannosylated, siRNA-delivering nanoparticles can induce immunostimulatory and tumor cytotoxic functions. Int J Nanomed.

[CR105] Paulus P, Stanley ER, Schäfer R (2006). Colony-stimulating factor-1 antibody reverses chemoresistance in human MCF-7 breast cancer xenografts. Cancer Res.

[CR106] Peng M, Huang B, Zhang Q (2014). Embelin inhibits pancreatic cancer progression by directly inducing cancer cell apoptosis and indirectly restricting IL-6 associated inflammatory and immune suppressive cells. Cancer Lett.

[CR107] Piaggio F, Kondylis V, Pastorino F (2016). A novel liposomal Clodronate depletes tumor-associated macrophages in primary and metastatic melanoma: anti-angiogenic and anti-tumor effects. J Control Release.

[CR108] Pienta KJ, Machiels J-P, Schrijvers D (2013). Phase 2 study of carlumab (CNTO 888), a human monoclonal antibody against CC-chemokine ligand 2 (CCL2), in metastatic castration-resistant prostate cancer. Investig New Drugs.

[CR109] Pitoia F, Jerkovich F (2016). Selective use of sorafenib in the treatment of thyroid cancer. Drug Des Dev Ther.

[CR110] Popivanova BK, Kostadinova FI, Furuichi K (2009). Blockade of a chemokine, CCL2, reduces chronic colitis-associated carcinogenesis in mice. Cancer Res.

[CR111] Priceman SJ, Sung JL, Shaposhnik Z (2010). Targeting distinct tumor-infiltrating myeloid cells by inhibiting CSF-1 receptor: combating tumor evasion of antiangiogenic therapy. Blood.

[CR112] Puig-Kröger A, Sierra-Filardi E, Domínguez-Soto A (2009). Folate receptor beta is expressed by tumor-associated macrophages and constitutes a marker for M2 anti-inflammatory/regulatory macrophages. Cancer Res.

[CR113] Pulaski HL, Spahlinger G, Silva IA (2009). Identifying alemtuzumab as an anti-myeloid cell antiangiogenic therapy for the treatment of ovarian cancer. J Transl Med.

[CR114] Pyonteck SM, Akkari L, Schuhmacher AJ (2013). CSF-1R inhibition alters macrophage polarization and blocks glioma progression. Nat Med.

[CR115] Qian BZ, Pollard JW (2010). Macrophage diversity enhances tumor progression and metastasis. Cell.

[CR116] Remon J, Girard N, Mazieres J (2016). Sunitinib in patients with advanced thymic malignancies: cohort from the French RYTHMIC network. Lung Cancer.

[CR117] Resnier P, Montier T, Mathieu V (2013). A review of the current status of siRNA nanomedicines in the treatment of cancer. Biomaterials.

[CR118] Ries CH, Cannarile MA, Hoves S (2014). Targeting tumor-associated macrophages with anti-CSF-1R antibody reveals a strategy for cancer therapy. Cancer Cell.

[CR119] Roberts AS, Campa MJ, Gottlin EB (2012). Identification of potential prognostic biomarkers in patients with untreated, advanced pancreatic cancer from a phase 3 trial (Cancer and Leukemia Group B 80303). Cancer.

[CR120] Rogers TL, Holen I (2011). Tumour macrophages as potential targets of bisphosphonates. J Transl Med.

[CR121] Rolny C, Mazzone M, Tugues S (2011). HRG inhibits tumor growth and metastasis by inducing macrophage polarization and vessel normalization through downregulation of PlGF. Cancer Cell.

[CR122] Rossi J-F, Négrier S, James ND (2010). A phase I/II study of siltuximab (CNTO 328), an anti-interleukin-6 monoclonal antibody, in metastatic renal cell cancer. Br J Cancer.

[CR123] Sandhu SK, Papadopoulos K, Fong PC (2013). A first-in-human, first-in-class, phase I study of carlumab (CNTO 888), a human monoclonal antibody against CC-chemokine ligand 2 in patients with solid tumors. Cancer Chemother Pharmacol.

[CR124] Segal NH, Gada P, Senzer N (2016). A phase II efficacy and safety, open-label, multicenter study of imprime PGG injection in combination with cetuximab in patients with stage IV KRAS-mutant colorectal cancer. Clin Colorectal Cancer.

[CR125] Shen J, Putt KS, Visscher DW (2015). Assessment of folate receptor-β expression in human neoplastic tissues. Oncotarget.

[CR126] Shi Y, Felder MA, Sondel PM (2015). Synergy of anti-CD40, CpG and MPL in activation of mouse macrophages. Mol Immunol.

[CR127] Shirota H, Klinman DM (2012). Effect of CpG ODN on monocytic myeloid derived suppressor cells. Oncoimmunology.

[CR128] Shirota Y, Shirota H, Klinman DM (2012). Intratumoral injection of CpG oligonucleotides induces the differentiation and reduces the immunosuppressive activity of myeloid-derived suppressor cells. J Immunol.

[CR129] Shirota H, Tross D, Klinman DM (2015). CpG oligonucleotides as cancer vaccine adjuvants. Vaccines.

[CR130] Shrivastava P, Singh SM, Singh N (2004). Activation of tumor-associated macrophages by thymosin alpha 1. Int J Immunopathol Pharmacol.

[CR131] Shrivastava P, Singh SM, Singh N (2005). Antitumor activation of peritoneal macrophages by thymosin alpha-1. Cancer Invest.

[CR132] Sica A, Mantovani A (2012). Macrophage plasticity and polarization: in vivo veritas. J Clin Investig.

[CR133] Sluijter M, van der Sluis TC, van der Velden PA (2014). Inhibition of CSF-1R supports T-cell mediated melanoma therapy. PLoS One.

[CR134] Smahel M, Duskova M, Polakova I (2014). Enhancement of DNA vaccine potency against legumain. J Immunother.

[CR135] Sośnicki S, Kapral M, Węglarz L (2016). Molecular targets of metformin antitumor action. Pharmacol Rep.

[CR136] Squadrito ML, Pucci F, Magri L (2012). miR-511-3p modulates genetic programs of tumor-associated macrophages. Cell Rep.

[CR137] Squadrito ML, Etzrodt M, De Palma M (2013). MicroRNA-mediated control of macrophages and its implications for cancer. Trends Immunol.

[CR138] Stafford JH, Hirai T, Deng L (2016). Colony stimulating factor 1 receptor inhibition delays recurrence of glioblastoma after radiation by altering myeloid cell recruitment and polarization. Neuro Oncol.

[CR139] Strachan DC, Ruffell B, Oei Y (2013). CSF1R inhibition delays cervical and mammary tumor growth in murine models by attenuating the turnover of tumor-associated macrophages and enhancing infiltration by CD8(+) T cells. Oncoimmunology.

[CR140] Su MJ, Aldawsari H, Amiji M (2016). Pancreatic cancer cell exosome-mediated macrophage reprogramming and the role of microRNAs 155 and 125b2 transfection using nanoparticle delivery systems. Sci Rep.

[CR141] Tang X, Mo C, Wang Y (2013). Anti-tumour strategies aiming to target tumour-associated macrophages. Immunology.

[CR142] Tian J, Ma J, Ma K (2013). β-Glucan enhances antitumor immune responses by regulating differentiation and function of monocytic myeloid-derived suppressor cells. Eur J Immunol.

[CR143] Van Acker HH, Anguille S, Willemen Y (2016). Bisphosphonates for cancer treatment: mechanisms of action and lessons from clinical trials. Pharmacol Ther.

[CR144] Vonderheide RH, Glennie MJ (2013). Agonistic CD40 antibodies and cancer therapy. Clin Cancer Res.

[CR145] Vonderheide RH, Flaherty KT, Khalil M (2007). Clinical activity and immune modulation in cancer patients treated with CP-870,893, a novel CD40 agonist monoclonal antibody. J Clin Oncol.

[CR146] Vonderheide RH, Bajor DL, Winograd R (2013). CD40 immunotherapy for pancreatic cancer. Cancer Immunol Immunother.

[CR147] Vonderheide RH, Burg JM, Mick R (2013). Phase I study of the CD40 agonist antibody CP-870,893 combined with carboplatin and paclitaxel in patients with advanced solid tumors. Oncoimmunology.

[CR148] Wang H, Shrestha TB, Basel MT (2012). Magnetic-Fe/Fe(3)O(4)-nanoparticle-bound SN38 as carboxylesterase-cleavable prodrug for the delivery to tumors within monocytes/macrophages. Beilstein J Nanotechnol.

[CR149] Wang Z, Sun J, Feng Y (2016). Oncogenic roles and drug target of CXCR4/CXCL12 axis in lung cancer and cancer stem cell. Tumor Biol.

[CR150] Wei Y, Schober A (2016). MicroRNA regulation of macrophages in human pathologies. Cell Mol Life Sci.

[CR151] Wu P, Nielsen TE, Clausen MH (2015). FDA-approved small-molecule kinase inhibitors. Trends Pharmacol Sci.

[CR152] Wu T, Dai Y, Wang W (2016). Macrophage targeting contributes to the inhibitory effects of embelin on colitis-associated cancer. Oncotarget.

[CR153] Xiang R, Luo Y, Niethammer AG (2008). Oral DNA vaccines target the tumor vasculature and microenvironment and suppress tumor growth and metastasis. Immunol Rev.

[CR154] Xin H, Zhang C, Herrmann A (2009). Sunitinib inhibition of Stat3 induces renal cell carcinoma tumor cell apoptosis and reduces immunosuppressive cells. Cancer Res.

[CR155] Yang J, Hawkins OE, Barham W (2014). Myeloid IKKb promotes antitumor immunity by modulating CCL11 and the innate immune response. Cancer Res.

[CR156] Zeisberger SM, Odermatt B, Marty C (2006). Clodronate-liposome-mediated depletion of tumour-associated macrophages: a new and highly effective antiangiogenic therapy approach. Br J Cancer.

[CR157] Zhan X, Jia L, Niu Y (2014). Targeted depletion of tumour-associated macrophages by an alendronate-glucomannan conjugate for cancer immunotherapy. Biomaterials.

[CR158] Zhang W, Zhu XD, Sun HC (2010). Depletion of tumor-associated macrophages enhances the effect of sorafenib in metastatic liver cancer models by antimetastatic and antiangiogenic effects. Clin Cancer Res.

[CR159] Zhang QQ, Hu XW, Liu YL (2015). CD11b deficiency suppresses intestinal tumor growth by reducing myeloid cell recruitment. Sci Rep.

[CR160] Zhu X, Fujita M, Snyder LA (2011). Systemic delivery of neutralizing antibody targeting CCL2 for glioma therapy. J Neurooncol.

